# Prescribing adrenaline auto-injectors in Slovenian children

**DOI:** 10.1186/2045-7022-5-S3-P109

**Published:** 2015-03-30

**Authors:** Tina Vesel, Anja Koren Jeverica, Metka Accetto, Natasha Toplak, Marijana Kuhar, Vesna Glavnik, Stefan Blazina, Gašper Markelj, Mirjana Zupančič, Mira Šilar, Peter Korošec, Tadej Avcin

**Affiliations:** 1 University Children's Hospital, University Medical Center, Ljubljana, Slovenia; 2 University Clinic of Respiratory and Allergic Diseases, Golnik, Slovenia

## Introduction

Little is known about the reasons for prescribing adrenalin auto-injectors in Slovenian children. Our objective was to evaluate prescription of adrenaline auto-injectors in children in our department in year 2013 and also to evaluate management of anaphylaxis of those children.

## Methods

We retrospectively collected data on interventions of anaphylaxis and allergic-clinic follow-up of 258 children which had adrenaline auto-injectors prescribed in year 2013.

## Results

Adrenaline auto-injector was prescribed in 258 children (66% boys and 34% girls). In 120 of them adrenaline auto-injector was prescribed de novo. 2% of them were babies, 31% 1 to 5 years old, 52% 6 to 14 years old and 15% 15 to 18 years old. In 66% adrenaline auto-injector was prescribed because of anaphylaxis, in 27% because of urticaria or/and angioedema and in 7% because of other reasons such as food allergy and bronchial asthma or/and recombinant based IgE testing results.

In 174 children (67%) adrenaline auto-injector was prescribed because of food allergy, most frequently because of allergy to peanuts (81 children), egg (35 children), tree nuts (19 children) and cow milk (14 children). 77% of peanut allergic children had IgE antibodies to rArah h 2. Among food allergic children 44% had multiple food allergies, 40% asthma and 11% suffered more than one immediate reaction. In 60 children (23%) adrenaline auto-injector was prescribed because of insect sting (49 had anaphylaxis and eleven urticaria or/and angioedema). Allergy to wasp or hornet venom was more frequently confirmed (in 34 children) than allergy to honeybee venom (23 children). 3 children had negative testing results after anaphylaxis due to a sting. In four children parents refused starting specific immunotherapy with Hymenoptera venom after anaphylaxis. In 12 children (5%) the cause of immediate reaction was unknown. Other confirmed reasons for prescribing adrenaline auto-injector were rare (such as cold, inhalant allergens, latex).

30% of anaphylaxis was treated with adrenaline (auto-injector was used in eight children). 23% of children were not admitted to hospital after anaphylaxis. No child was admitted to intensive care unit.

## Conclusions

Our data showed that allergy to peanuts was most frequent cause of prescribing adrenaline auto-injector to children during last year. There is need to increase education of management of anaphylaxis in children in Slovenia.

